# Poison Sumac

**Published:** 1882-01

**Authors:** 


### THE POISON SUMAC.

This sumac is terrible in its effects, often causing temporary blindness. Years ago it became the fashion to wear immense wreaths and bunches of artificial flowers inside and outside of ladies' bonnets. The flower makers, being hard pressed for material, made use of dried grasses, seed-vessels, burs, and catkins; these were painted, dyed, frosted and bronzed, to make them attractive. I became greatly interested in the business and the ingenuity displayed, and spent much time examining the contents of milliners' windows. On one occasion, when standing before a very fashionable milliner's window on Fourteenth street, I was horror-stricken on discovering that an immense wreath of grayish berries, which constituted the inside trimming of a bonnet, was composed entirely of the berries of the poison sumac, just as they had been gathered, not a particle of varnish, bronze, or other material coating them. The bonnet, when worn, would bring the entire mass of villainous berries on the top and sides of the head, and a few of the sprays about the ears and on the forehead. Stepping into the store, I addressed the proprietor and asked her if she knew that the bonnet was trimmed with the berries of one of the most poisonous shrubs known in the United States. After staring at me in a sort of puzzled way, she informed me that I was mistaken; that she had received those flowers from Paris only a week ago. "Madam," I replied, "there must be a mistake somewhere, for those are the berries of the poison sumac, which does not grow in Europe." She gave me one angry look, asked me to please attend to my own business, and swept away from me to the other end of the store. A few days after this I read in the daily papers an account of the poisoning of a number of small girls employed in a French artificial flower manufactory in Green street. I at once guessed the cause. I visited the factory mentioned, introduced myself to the proprietor, told him what I knew about the poison berries—and was rudely requested to make myself scarce. After these two adventures I made up my mind to keep my botanical knowledge (poisonous though it might be) to myself.—*Harper's Young People.*

---

THE HOUSEHOLD RECIPE.—In the information column of some of our exchanges, the startling assertion that a watermelon may be varnished and will keep until Christmas is still going around.

The watermelon is our favorite fruit, and we thought last fall that we would varnish and surprise our friends with it Christmas. The surprise was all right, but the melon wasn't good for much. After the turkey and cranberry sauce had been demolished, we remarked, as we sharpened the carving knife on our boot leg, that we would now deal out the fruit.

When we shoved the glittering blade through the varnished shell of the watermelon, about a quart of melon juice in a bad state of preservation squirted around the festive board and stopped the flow of conversation.

You can't always bet on these recipe that you read in the papers. We remember once of reading that a certain preparation would keep eggs for a year, and they would taste better when you peeled them than when they were first picked.

We tried it.

They seemed filled with malaria when we opened them.—*Laramie Boomerang.*

A TELEGRAPH STORY.—One of the operators in the Western Union office at Boston had an epileptic fit. His medical attendant spoke to him, chafed him, and made every effort to arouse him, but in vain. Subsequently one of his fellow-operators drew a chair up to the bed, and took the patient's hand in his. As he did so, he noticed a feeble pressure by the fingers, which pressure presently resolved itself into dots and dashes, faintly communicating to the tactile sense the words: 'W-h-a-t d-o-c-t-o-r s-a-y a-b-o-u-t m-e ?" Asked whether he could hear what was said to him, the patient signified assent by a slight motion with the tips of his fingers, and the result was that his fellow-operator got from the patient enough dots and dashes to describe his feelings to the physician, who was thus enabled to apply the necessary remedies. It is certain that no other method of communication was possible under the circumstances, since the sufferer from epilepsy, although he could hear, could neither speak nor move any of his muscles, except those situated in the digital extremities, and those only with the faintest requisite in electrical communication.

LAURA B. M., Kaufman.—" I hear a great deal about a new fashionable folly—the decoration of plates by amateur artists. How is the thing done, and can you describe what the decoration consists of ?"

We have had several plates decorated lately by female members of our family. The way they did it was after this fashion : They first warmed the plate, then they laid on it several slices of the breast of a turkey, a second joint, some cranberries, dressing (without onions), and a couple of boiled Irish potatoes. Try that sort of decoration, and your friends will appreciate your artistic ability.—*Texas Siftings.*

PROFESSOR HUXLEY says : If the sound of music doesn't cause a dog acute pain, why does the animal sit up on its haunches and howl when a German band is doing its worst in the street ?" We'll tell you, professor. The dog acts that way because it loves music, and is waiting for an opportunity to grab the leader of the band by the throat. It is not a sign that a dog doesn't love music because a street band makes him howl. Old Mendelssohn would sit upon his hind legs and howl, too, if he could hear some of the street music of our day.—*The Judge.*

EXPECTANCY OF LIFE.—Insurance companies are aware of the incredulous weakness of those whose lives they assure, and have therefore compiled numerous tables of expectancy of life for their own guidance, which are carefully referred to before a policy is granted. These tables have been the result of careful calculation, and seldom prove misleading. Of course, sudden and premature deaths, as well as lives unusually extended, occasionally occur, but the average expectancy of life of an ordinary man or woman is as follows: A person 1 year old may expect to live 39 years longer; of 10 years, 51; of 20 years, 41; of 30 years, 34; of 40 years, 28; of 50 years, 21; of 60 years, 14; of 70 years, 9; of 80 years, 4.

Experimental measurements of the temperature of the body have led M. E. Villari to the conclusion that the lowest temperature in man, ensuing after a period of rest, is 98.4 degrees; that the temperature increases, under the influence of a positive, ascending effort, to 100.6 degrees, under the influence of a descending effort to 100.3 degrees; that it increases after any exertion, but more after an ascending than after a descending one; and that the chemical actions of the organism are augmented after every movement.

To Kill a Felon.—To kill a felon, or bring it to a head if too far advanced to kill, take poke root and boil till soft enough to make a poultice, apply, and if the felon is not too far advanced it will scatter it; if it is, it will hasten suppuration. It will deaden the pain in a short time and save much suffering. I have used this myself and know it to be good.

Salt Food.—The excessive use of salt food will produce salt-rheum and the canker, and also affect the hands, causing roughness and cracking. Corn huskers have need to be careful with their hands; they should do without soap as much as possible, and grease the affected parts at night with unsalted mutton fat well warmed and rubbed in.

A Sure Cure for Chilblains.—Boil one pound of alum till dissolved, place the feet in the water as hot as it can be borne, and keep there till the alum has solified. Two applications cured the worst case I ever knew. Mine were disposed of effectually at one trial.

Great ideas travel slowly and for a time noiselessly, as the gods whose feet were shod with wool.

